# Radioactive artificial ^137^Cs and natural ^40^K activity in 21 edible mushrooms of the genus *Boletus* species from SW China

**DOI:** 10.1007/s11356-017-8494-7

**Published:** 2017-02-01

**Authors:** Jerzy Falandysz, Ji Zhang, Tamara Zalewska

**Affiliations:** 1 Gdańsk University, 63 Wita Stwosza Str, 80-803 Gdańsk, Poland; 2 Institute of Medicinal Plants, Yunnan Academy of Agricultural Sciences, 2238 Beijing Road, Panlong District, Kunming, 650200 China; 3 Yunnan Technical Center for Quality of Chinese Materia Medica, Kunming, 650200 China; 4 Institute of Meteorology and Water Management, National Research Institute, Maritime Branch, Waszyngtona 42, 81-342 Gdynia, Poland

**Keywords:** Asia, Yunnan, Organic food, Radioactive fallout, Wild mushrooms

## Abstract

This study, for the first time, presents the results of activity concentration determinations for ^137^Cs and ^40^K in a high number (21 species, 87 composite samples, and 807 fruiting bodies) of mushrooms of the genus *Boletus* from across Yunnan in 2011–2014 and Sichuan (*Boletus tomentipes*) using high-resolution high-purity germanium detector. Activity concentrations of ^137^Cs demonstrated some variability and range from <4.4 to 83 ± 3 Bq kg^−1^ dry biomass in caps and from <3.8 to 37 ± 3 Bq kg^−1^ dry biomass in stipes, and of ^40^K, respectively, from 420 ± 41 to 1300 ± 110 and from 520 ± 61 to 1300 ± 140 Bq kg^−1^ dry biomass. No significant variations were observed regarding ^137^Cs and ^40^K activity concentrations among the same *Boletus* species from different sampling sites. No activity concentrations from ^134^Cs were detected in any mushrooms. Internal dose rates estimated were from intake of 1 kg of mushrooms per annum for ^137^Cs range for species and regions from around <0.0031 to 0.047 ± 0.003 μSv, while those for ^40^K were from around 0.22 ± 0.04 to 1.2 ± 0.1 μSv. The overall intake of ^137^Cs was low since low contamination was found in *Boletus* species.

## Introduction

The nuclear weapon detonations in the atmosphere (1945–1980) and two major nuclear power plant accidents in Chernobyl (1986) and in Fukushima (2011) caused radioactive fallout at a global scale but deposition rates, radioactive pollution, and health risk differed for the regions of the world (Haselwandter et al. 1988; Marzo 2014; Steinhauser et al. 2013 and 2014). The long-term residual radioactivity in the affected areas after the nuclear weapons use and nuclear power plant accidents comes largely from radiocaesium (^137^Cs, half-life 30.05 years, and ^134^Cs, half-life 2.06 years) aerosol deposited onto soils (Yasunari et al. 2011). The Chernobyl accident not only affected land mostly in the Central and Northern Europe and especially in the Ukraine, Belarus, and Russia (De Cort et al. 1998), but also with health consequences (thyroid cancer development, because of high exposure for radioactive iodine from Chernobyl) in Poland.

Mushrooms are effective in bioconcentration in fruiting bodies of various metallic elements, semimetals, and radionuclides absorbed by mycelium from soil substrata (Baeza et al. 2005; Falandysz et al. 2007, 2011, 2014; Kalač 2001; Kojta and Falandysz 2016; Marzano et al. 2001), and many species can be highly contaminated by accumulated ^137^Cs which was deposited onto forests localized close or more distantly from the source of emission (Grodzinskaya et al. 2003, 2013).

The number of the pre-Chernobyl data published on activity concentrations of ^137^Cs accumulated in mushrooms because of the nuclear weapons tests in the atmosphere is a few. Mushrooms such as *Cortinarius armillatus*, *Cortinarius caperatus*, *Paxillus involutus*, *Lactarius rufus*, *Suillus grevillei*, *Cantharellus cibarius*, and *Amanita rubescens* collected from the western Austria in 3 to 5 months after the Chernobyl accident showed ^137^Cs in fruiting bodies in activity concentrations from 3-fold to 4.8-fold greater than before the catastrophe (Haselwandter et al. 1988). Also, a pre-Chernobyl data on concentration activity of ^137^Cs in mushrooms are available for the territory of Poland. A valued *Boletus edulis* from the pre-Chernobyl collection (1984 and 1985) from a place located circa 650 km west of the feral nuclear power plant site in Poland showed ^137^Cs in fruit bodies at 95–104 Bq kg^−1^ dry biomass (db), while contamination was 2.5-fold to 4-fold greater in 1986–1988 (Bem et al. 1990).

Mushrooms differ in accumulation capacity of ^137^Cs in fruiting bodies, and this feature is highly determined by the species-specific status of the stabile caesium (^133^Cs) on one side and a degree of environmental (litter and/or soil horizons) pollution with ^137^Cs on the other, and what results also in a positive correlation between the activity concentrations in forest topsoil and mushrooms (Falandysz and Borovička 2013; Falandysz et al. 2015a; Yoshida and Muramatsu 1998; Yoshida et al. 2000, 2004).

The Chernobyl accident caused substantial contamination with radiocaesium of wild-growing mushrooms in the Central and Northern Europe and especially in the Ukraine, Belarus, and Russia (Falandysz et al. 2015a; Grodzinskaya et al. 2003 and 2013; Smith et al. 1993; Taira et al. 2011). However, locally or regionally, also mushrooms form some other locations in Europe were highly affected (Mietelski et al. 2010; Strandberg 2004; Zalewska et al. 2016), while in many other sites were much less or little affected (Daillant et al. 2013; García et al. 2015; Karadeniz and Yaprak 2010; Rakić et al. 2014; Turhan et al. 2007). From the toxicological point of view, some radioactive compounds other than radiocaesium accumulated by fungi in fruiting bodies can also highly matter, but the number of published data is much less (Kirchner and Daillant 1998; Saniewski et al. 2016; Strumińska-Patulska et al. 2016).

The Chernobyl nuclear power plant accident had little effect in China (Pang et al. 1989; Wang et al. 1998). Also, the Fukushima Dai-ichi nuclear power plant accident had little if any effect in China (Liu et al. 2013; Povinec et al. 2013; Shuai et al. 2016; Wan et al. 2014). Nevertheless, an internationally available data on radioactive fallout from the nuclear weapons tests and nuclear plants accidents are highly limited from China so far. Also, no data inventory on the surface contamination with ^134/137^Cs (Bq per m^2^) is available from the Yunnan province yet.

In Yunnan is a high biodiversity of mushrooms of the genus *Boletus* and many are unique for this region of the world (Wu et al. 2016). Yunnan Province is the major supplier of wild-growing fungi in mainland China and for export. Also, it is a great tradition for foraging and eating fungi there. In Yunnan, as reported for the individuals from over 2 million people of the Liangshan Yi nationality, an annual rate of wild-grown fungi consumption could locally even exceed 20–24 kg per capita (Zhang et al. 2010). This study aimed to provide and discuss information on radiocaesium (^134/137^Cs) and radioactive potassium (^40^K) accumulated and distributed in fruiting bodies by 22 species of fungi of the genus *Boletus*, which are highly valued organic food (Frankowska et al. 2010) and which are widely foraged in Yunnan of China. We aimed also to examine if there are variations in the nuclide activity among the individuals of the same species collected from the spatially distantly distributed sites and between fruit bodies of different *Boletus* species which are grown in the same area in Yunnan.

## Materials and methods

**Fig. 1:**
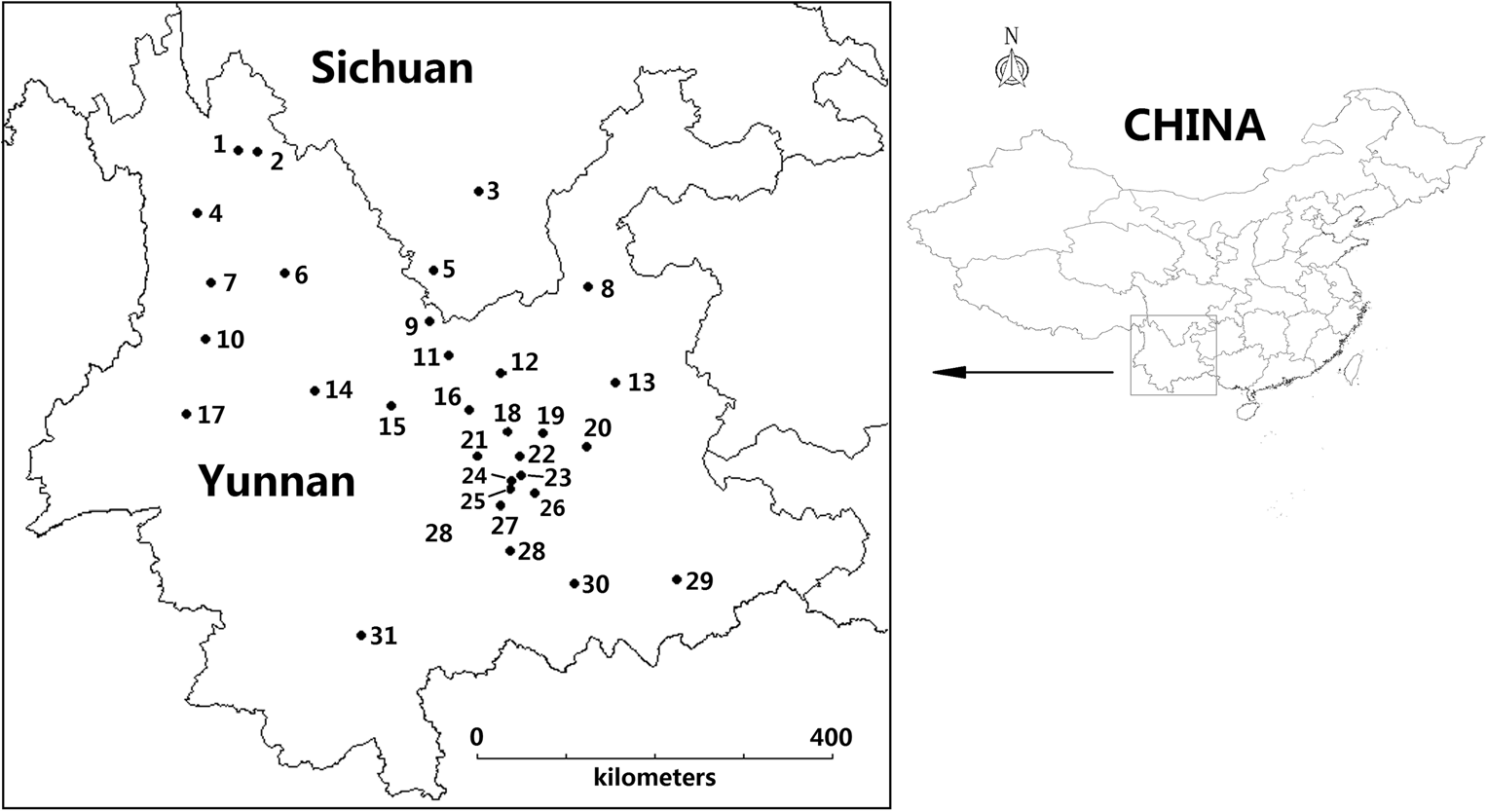
Localization of the sampling sites (1–31 ; for details, see Table 1)

In order to investigate fungi of the genus *Boletus* representative of Yunnan, we chose 21 species representing widely distributed locations and collected several individuals from a species in a given location, which were combined into composite samples (Fig. 1). Fungi collected in July to September 2011–2014 in Yunnan and in July to August 2012 in Sichuan in this study include *Boletus aereus* Fr. ex Bull., *Boletus auripes* Peck, *Boletus bicolor* Peck, *Boletus brunneissimus* Chiu, *Boletus calopus* Fr., *B. edulis* Fr., *Boletus erythropus* Fr., *Boletus ferrugineus* Schaeff, *Boletus fulvus* Peck, *Boletus griseus* Frost., *Boletus impolitus* Fr., *Boletus luridus* Fr., *Boletus magnificus* Chiu., *Boletus obscureumbrinus* Hongo, *Boletus pallidus* Frost., *Boletus purpureus* Fr. (current name *Imperator rhodopurpureus* Smotl.), common name purple bolete), *Boletus reticuloceps* M. Zang, M.S. Yuan & M.Q. Gong Q.B. Wang & Y.J. Yao, *Boletus sinicus* W.F. Chiu, *Boletus speciosus* Frost., *Boletus tomentipes* Earle, and *Boletus umbriniporus* Hongo (Index Fungorum, 2016; Mao 2009).

Each fruit body was separated into cap (with skin) and stipe in order to examine the distribution of ^137^Cs and ^40^K between its morphological parts. The collected fruit bodies were in good “edible” body condition (not injured by insects) and well developed. The individual cap and stipe samples were sliced into pieces using a disposable plastic knife and pooled for each site (*n* = 5 to 21 individuals per pool)—with composite samples representing each species, sampling location, and time of collection (Table 1). Subsequently, the fungal samples were dried in plastic shelves of a commercial electric dryer (model: Ultra FD1000 dehydrator, Ezidri, Australia) at 65 °C to constant mass. Dried fungal materials were pulverized in a porcelain mortar(s) using a porcelain pestle(s) that was cleaned using a laboratory brush, deionized water, and detergent and further rinsed with distilled water and dried in an electrically heated laboratory dryer at 105 °C. The samples were kept in sealed polyethylene bags under dry conditions. The pooled fungal materials mass obtained and used in this study was in the range 25∼200 g dry biomass—it differed per species, morphological part, and localization, because of a different size and quantity of the fruiting bodies available per sample.

**Table 1:** ^137^Cs and ^40^K in mushrooms of the genus *Boletus* species from the Yunnan and Sichuan provinces, China (Bq kg^−1^ db; activity concentration ± measurement uncertainty)

Species, location^*^, and year of collection	Number	^137^Cs		^40^K	
		Fruiting bodies		Fruiting bodies	
		Caps	Stipes	Caps	Stipes
*Boletus aereus* Fr. ex Bull					
[9]* Yongren, Chuxiong; 2012	(10)**	<4.8	<3.9	1200 ± 130	830 ± 100
[29] Dongshan, Wenshan; 2012	(7)	22 ± 4	11 ± 2	1600 ± 300	870 ± 150
*Boletus auripes* Peck					
[21] Yimen, Yuxi; 2011	(9)	7.9 ± 2.1	5.8 ± 1.7	1100 ± 140	620 ± 120
[12] Wuding, Chuxiong; 2011	(11)	11 ± 2	8.7 ± 1.6	1400 ± 170	840 ± 160
*Boletus bicolor* Peck					
[26] Jiangchuan, Yuxi; 2012	(10)	8.3 ± 1.6	8.5 ± 1.7	1100 ± 110	1200 ± 110
[25] Dayingjie, Yuxi; 2013	(7)	5.2 ± 1.7	4.9 ± 1.1	630 ± 140	470 ± 91
[25] Dayingjie, Yuxi; 2014	(15)	21 ± 3	10 ± 2	1300 ± 200	1400 ± 260
*Boletus brunneissimus* Chiu					
[18] Anning, Kunming; 2012	(9)	13 ± 2	5.4 ± 1.4	1200 ± 150	1000 ± 97
*Boletus calopus* Fr					
[13] Malong, Qujing; 2013	(10)	6.8 ± 1.1	3.8 ± 0.9	1000 ± 82	770 ± 75
*Boletus edulis* Bull: Fr					
[22] Jinning, Kunming; 2011	(11)	4.8 ± 3.4	9.4 ± 1.9	1100 ± 140	900 ± 121
[18] Anning, Kunming; 2012	(10)	14 ± 2	11 ± 2	WD	WD
[20] Shilin, Kunming; 2012	(10)	13 ± 1	9.3 ± 1.4	1400 ± 110	1000 ± 94
[21] Yimen, Yuxi; 2011	(12)	10 ± 1	5.0 ± 1.0	740 ± 86	360 ± 61
[21] Yimen, Yuxi; 2012	(10)	5.4 ± 1.2	5.5 ± 1.2	810 ± 74	500 ± 65
[21] Yimen, Yuxi; 2012	(10)	8.2 ± 1.5	4.6 ± 1.1	1100 ± 92	1200 ± 72
[15] Nanhua, Chuxiong; 2011	(7)	9.6 ± 2.5	7.1 ± 2.1	1500 ± 170	840 ± 140
[15] Nanhua, Chuxiong; 2011	(10)	4.7 ± 1.4	11 ± 2	1200 ± 120	1100 ± 120
[15] Nanhua, Chuxiong; 2013	(10)	3.6 ± 10	<2.4	WD	WD
[14] Midu, Dali; 2012	(10)	10 ± 2	13. ± 2	1200 ± 110	860 ± 98
[6] Heqing, Dali; 2012	(7)	4.7 ± 1.4	<3.3	1300 ± 110	850 ± 89
[17] Longyang region, Baoshan; 2012	(10)	7.5 ± 1.4	4.0 ± 0.9	WD	WD
[29] Dongshan, Wenshan; 2012	(7)	<4.5	<3.2	1200 ± 120	930 ± 84
*Boletus erythropus* Fr.					
[13] Malong, Qujing; 2013	(5)	8.3 ± 1.6	6.2 ± 1.5	1000 ± 100	860 ± 140
*Boletus ferrugineus* Schaeff					
[2] Pudacuo, Diqing; 2012	(10)	14 ± 3	WD	960 ± 110	WD
[7] Lanping, Nujiang; 2012	(7)	15 ± 1	20 ± 1	420 ± 41	520 ± 61
[12] Wuding, Chuxiong; 2011	(10)	<3.7	<3.8	1200 ± 120	1000 ± 130
[19] Kunming city; 2011	(8)	<3.3	<3.2	1100 ± 100	930 ± 100
[20] Shilin, Kunming; 2012	(9)	5.6 ± 1.4	3.9 ± 1.3	730 ± 63	690 ± 63
[21] Yimen, Yuxi; 2012	(9)	8.2 ± 2.1	12 ± 2	880 ± 130	710 ± 110
*Boletus fulvus* Peck					
[14] Midu, Dali; 2012	(10)	9.1 ± 1.8	5.2 ± 1.4	1000 ± 110	800 ± 99
*Boletus griseus* Frost.					
[19] Kunming city; 2011	(8)	7.3 ± 1.6	16 ± 2	1600 ± 120	1500 ± 150
[18] Anning, Kunming; 2012	(7)	15 ± 2	<4.8	1300 ± 140	1400 ± 130
[20] Shilin, Kunming; 2012	(10)	5.5 ± 1.5	<3.5	1700 ± 140	1400 ± 93
[17] Longyang region, Baoshan; 2012	(10)	8.1 ± 1.7	8.7 ± 1.6	1800 ± 130	1700 ± 120
[26] Jiangchuan, Yuxi; 2012	(10)	6.8 ± 1.5	<4.9	1500 ± 120	1300 ± 120
[14] Midu, Dali; 2012	(10)	9.6 ± 1.9	<5.0	1500 ± 130	1500 ± 130
[13] Malong, Qujing; 2012	(7)	<4.8	13 ± 2	1400 ± 130	1300 ± 140
[16] Lufeng, Chuxiong; 2013	(10)	5.8 ± 1.1	4.0 ± 1.0	1500 ± 85	1200 ± 83
*Boletus impolitus* Fr.					
[19] Kunming city; 2011	(10)	8.0 ± 1.5	13 ± 2	1400 ± 110	880 ± 120
[21] Yimen, Yuxi; 2012	(8)	11 ± 2	15 ± 2	1100 ± 120	930 ± 130
*Boletus luridus* Schaoff.:Fr.					
[11] Yuanmou, Chuxiong; 2012	(8)	9.9 ± 1.9	11 ± 2	1500 ± 140	860 ± 130
[14] Midu, Dali; 2012	(8)	7.4 ± 1.7	9.2 ± 1.7	1500 ± 120	1300 ± 120
[20] Shilin, Kunming; 2012	(8)	36 ± 2	10 ± 1	1000 ± 120	240 ± 79
*Boletus magnificus* Chiu.					
[26] Jiangchuan, Yuxi; 2012	(10)	3.9 ± 1.2	<4.0	1100 ± 120	900 ± 100
[21] Yimen, Yuxi; 2012	(10)	6.1 ± 1.6	5.9 ± 1.2	1400 ± 110	740 ± 73
[21] Yimen, Yuxi; 2012	(7)	<5.2	9.8 ± 1.8	1500 ± 140	960 ± 180
[14] Midu, Dali; 2012	(9)	5.7 ± 1.3	WD	830 ± 89	WD
[25] Dayingjie, Yuxi; 2014	(8)	8.8 ± 1.6	8.3 ± 1.6	1500 ± 120	1100 ± 110
*Boletus obscureumbrinus* Hongo					
[31] Simao region, Puer; 2013	(10)	11 ± 2	<4.7	1600 ± 130	1500 ± 120
[31] Simao region, Puer; 2013	(21)	15 ± 2	7.5 ± 1.6	1400 ± 150	1400 ± 110
*Boletus pallidus* Frost.					
[18] Anning, Kunming; 2012	(10)	9.9 ± 1.9	<5.0	1700 ± 130	1000 ± 130
*Boletus purpureus* Fr.					
[21] Yimen, Yuxi; 2012	(10)	6.8 ± 1.6	5.7 ± 1.7	WD	WD
[21] Yimen, Yuxi; 2012	(8)	9.0 ± 1.7	8.5 ± 2.1	1900 ± 140	1700 ± 130
[10] Yunlong, Dali; 2012	(10)	12 ± 2	6.1 ± 1.9	1300 ± 170	870 ± 140
[7] Lanping, Nujiang; 2012	(12)	6.2 ± 1.7	9.7 ± 1.9	1200 ± 110	840 ± 110
*Boletus reticuloceps* Zang et al.					
[2] Pudacuo, Diqing; 2012	(10)	13 ± 1	9.9 ± 1.8	1200 ± 77	700 ± 97
*Boletus sinicus* W.F. Chiu					
[13] Malong, Qujing; 2013	(10)	6.3 ± 1.5	6.6 ± 1.4	1500 ± 110	1200 ± 92
[24] Jiulongchi, Yuxi; 2013	(8)	7.2 ± 1.6	3.2 ± 0.9	1400 ± 97	1100 ± 67
*Boletus speciosus* Frost.					
[18] Anning, Kunming; 2012	(10)	<5.5	7.9 ± 1.4	1000 ± 120	730 ± 87
[18] Anning, Kunming; 2012	(10)	21 ± 3	9.9 ± 1.8	1500 ± 170	1000 ± 110
[21] Yimen, Yuxi; 2012	(10)	6.8 ± 1.5	9.4 ± 1.7	1100 ± 110	730 ± 110
[17] Longyang region, Baoshan; 2012	(10)	14 ± 3	9.5 ± 1.6	1400 ± 170	840 ± 110
[11] Yuanmou, Chuxiong; 2012	(10)	6.1 ± 1.3	4.7 ± 1.1	1100 ± 86	690 ± 72
[8] Huize, Qujing; 2013	(10)	6.6 ± 1.5	<2.6	1000 ± 94	750 ± 69
*Boletus tomentipes* Earle					
[27] Eshan, Yuxi; 2011	(10)	<4.4	9.2 ± 1.7	1600 ± 110	1400 ± 100
[27] Eshan, Yuxi; 2011	(9)	8.5 ± 1.6	4.2 ± 1.3	1200 ± 100	1200 ± 98
[27] Eshan, Yuxi; 2011	(7)	6.9 ± 1.2	4.5 ± 1.2	1300 ± 110	1200 ± 81
[21] Yimen, Yuxi; 2011	(10)	9.0 ± 1.9	9.7 ± 1.7	1600 ± 120	1300 ± 100
[21] Yimen, Yuxi; 2011	(9)	5.8 ± 1.4	9.8 ± 1.7	1200 ± 99	1300 ± 110
[31] Simao region, Puer; 2011	(10)	14 ± 2	11 ± 2	1900 ± 140	1400 ± 120
[15] Nanhua, Chuxiong; 2011	(8)	7.1 ± 1.4	9.7 ± 1.8	1200 ± 120	1300 ± 120
[30] Gejiu, Honghe; 2012	(7)	14 ± 2	5.4 ± 1.2	1500 ± 110	1300 ± 88
[1] Shangri-la, Diqing; 2011	(9)	6.2 ± 1.3	6.6 ± 1.5	1400 ± 88	1300 ± 98
[28] Shiping, Honghe; 2012	(8)	7.0 ± 1.6	3.3 ± 0.8	1300 ± 100	940 ± 72
[3] Dechang, Sichuan; 2012	(9)	4.8 ± 1.6	8.3 ± 1.3	1800 ± 110	1800 ± 100
[5] Panzhihua, Sichuan; 2012	(10)	5.3 ± 1.3	9.0 ± 1.5	1500 ± 96	1600 ± 95
[6] Heqing, Dali; 2012	(7)	5.2 ± 1.1	4.3 ± 1.1	1100 ± 86	1000 ± 75
*Boletus umbriniporus* Hongo					
[21] Yimen, Yuxi; 2011	(10)	4.9 ± 1.4	10 ± 2	1100 ± 120	630 ± 140
[21] Yimen, Yuxi; 2012	(7)	14 ± 2	7.2 ± 1.6	1500 ± 150	1300 ± 140
[23] Huangcaoba, Yuxi; 2011	(8)	6.4 ± 1.2	<3.6	910 ± 87	910 ± 93
[17] Longyang region, Baoshan; 2012	(7)	6.5 ± 1.3	7.8 ± 1.8	1400 ± 91	960 ± 110
[14] Midu, Dali; 2012	(8)	13 ± 1	<3.9	1300 ± 100	1100 ± 100
[28] Shiping, Honghe; 2012	(9)	7.7 ± 1.5	8.7 ± 1.4	990 ± 98	660 ± 83
[11] Yuanmou, Chuxiong; 2011	(10)	9.3 ± 1.7	16 ± 2	1300 ± 110	1200 ± 120
[8] Huize, Qujing; 2013	(10)	8.1 ± 1.4	7.2 ± 1.3	1200 ± 91	880 ± 82
[15] Nanhua, Chuxiong; 2013	(10)	8.4 ± 1.4	5.2 ± 1.0	1200 ± 91	930 ± 73

** Number of individuals

*WD* without data

^*^See Fig. 1

Activity concentrations of ^137^Cs and ^40^K were determined using a gamma spectrometer with coaxial HPGe detector with a relative efficiency of 18% and a resolution of 1.9 keV at 1.332 MeV (with associated electronics) (Falandysz et al. 2016; Zalewska and Saniewski 2011). The equipment was calibrated using a standard mixture of gamma-emitting isotopes of different elements, and the method was fully validated. The reference solution, “Standard solution of gamma emitting isotopes, code BW/Z-62/27/07” produced by IBJ-Świerk near Otwock in Poland, was used for preparing reference samples for the equipment calibration. The same geometry of cylindrical dishes with 40 mm diameter (as applied for environmental samples) was used for reference samples during equipment calibration. Also calculated were the values of the ^137^Cs and ^40^K cap to stipe concentration quotients (index Q_c/s_).

The laboratory involved was subjected to routine checks to ensure high standards of analytical quality and analytical control and participated successfully in the inter-comparison exercises organized by IAEA-MEL Monaco (IAEA-414, Irish and North Sea Fish) to confirm the reliability and accuracy of the method (Zalewska and Saniewski 2011). Repeated analysis gave values of ^137^Cs, 5.06 ± 0.64 Bq kg^−1^ db, and ^40^K, 474.5 ± 19.3 Bq kg^−1^ db, while the estimated target values were equal to 5.18 ± 0.10 Bq kg^−1^ db for ^137^Cs and 481 ± 16 Bq kg^−1^ db for ^40^K. In the gamma spectrometry measurements, the limit of quantification was calculated using GENIE 2000 as a minimum detectable activity (MDA) as defined by Curie (1968). All numerical data gained were recalculated for dehydrated fungal material (at 105 °C), and all data were decay corrected back to the time of sampling.

## Results and discussion

### ^137^Cs activity concentrations in *Boletus* species

The activity concentrations of ^137^Cs in fungi of the genus *Boletus* species from the Yunnan and Sichuan showed low contamination: for the caps, total range was between <3.3 and 36 ± 2 Bq kg^−1^ db and for stipes was between <2.4 and 20 ± 1 Bq kg^−1^ db (Table 1). No activity from ^134^Cs was detected at the time of measurements (summer 2015) in any sample in this study. The *Boletus* fungi usually showed detectable activity concentrations, but in the case of a several collected composite samples of different species, the ^137^ Cs activity concentrations were below the limit of determination: in detail, in seven composite samples of the caps (<3.3–<5.2 Bq kg^−1^ db) and 15 samples of the stipes (<2.6–5.0 Bq kg^−1^ db).

The median value of the activity concentrations of ^137^Cs in the caps for a whole collection of *Boletus* spp. (87 composite samples with data) was at 7.7 Bq kg^−1^ db, and the arithmetic mean and standard error were at 8.7 ± 5.1 Bq kg^−1^ db [if activity concentration in a sample was below the value of the method limit of detection (LOD), a half of this value was used in calculations]. The median value of the activity concentration of ^137^Cs in the stipes for a whole collection of *Boletus* spp. (85 composite with data) was at 7.2 Bq kg^−1^ db, and the arithmetic mean and standard error were at 7.1 ± 3.2 Bq kg^−1^ db. The median value of the index Q_C/S_ for ^137^Cs in fruit bodies of all the *Boletus* fungi with data (64 composite samples) was 1.1 (the arithmetic mean and standard error values were at 1.3 ± 0.5 and range 0.42–3.6).

The activity concentrations of ^137^Cs in the composite samples of a particular species of the *Boletus* fungi with data for more than three sample sets were roughly similar, e.g., in the caps, the ranges were as follows: <4.5–14 ± 2 Bq kg^−1^ db for *B. edulis*, <3.3–15 ± 1 Bq kg^−1^ db for *B. ferrugineus*, <4.8–15 ± 2 Bq kg^−1^ db for *B. griseus*, <5.2–8.8 ± 1.6 Bq kg^−1^ db for *B. magnificus*, 6.2 ± 1.7–12 ± 2 Bq kg^−1^ db for *B. purpureus*, <5.5–21 ± 3 Bq kg^−1^ db for *B. speciosus*, <4.4–14 ± 2 Bq kg^−1^ db for *B. tomentipes*, and 6.4 ± 1.2–14 ± 2 Bq kg^−1^ db for *B*. *umbriniporus.*

Fungus *B. luridus*, for which data were available from only three locations, showed maximal value of the activity concentrations of ^137^Cs determined in the caps in this study, and range was 7.4 ± 1.7–36 ± 2 Bq kg^−1^ db (Table 1). Is the question of matter if this can be related to differences in soil pollution with ^137^Cs, which is doubtful because of a narrow range and low activity concentrations for almost all sample sets (Table 1) or more to better (species-specific or site-specific) bioconcentration potential of ^137^Cs by *B. luridus* than other fungi examined? A major reason for a species-specific difference in susceptibility of fungi to contamination with radiocaesium can be related to the status of stable Cs (^133^Cs). Certain species of fungi are richer in stable Cs than other species under the same field conditions, and uptake of ^137^Cs follows well an uptake of ^133^Cs (Falandysz and Borovička 2013; Yoshida et al. 2000, 2004).

The composite sample of *B. luridus* from the Shilin site in the Kunming region, which showed activity concentration of ^137^Cs in the caps at 36 ± 2 Bq kg^−1^ db and in the stipes at 10 ± 1 Bq kg^−1^ db, contained the total Cs (including ^137^Cs) in the caps at 160 mg kg^−1^ db and in the stipes at 38 mg kg^−1^ db (Saba et al. 2016). Two other samples of *B. luridus*, e.g., from the Midou and Yuanmou sites, contained ^137^Cs in the caps and stipes respectively at 7.4–9.9 and 9.7–11 Bq kg^−1^ db, while total Cs in the caps was at 0.65–3.0 mg kg^−1^ db and in the stipes 0.37–2.4 mg kg^−1^ db. A positive relationship between stable ^133^Cs and radioactive ^137^Cs in examined *B. luridus* seems possible.

As mentioned earlier, there are a few data published on the activity concentrations of ^134^Cs and ^137^Cs in fungi foraged in Yunnan and no data for other regions of the mainland China. In one of our previous studies, it was shown that the activity concentrations of ^137^Cs in sclerotia of the fungus *Wolfiporia extensa* collected across Yunnan were low, i.e., in range <1.4 to 7.2 ± 1.1 Bq kg^−1^ db (^134^Cs was undetected) (Wang et al. 2015). Similarly, also, the pantropical fungus *Macrocybe gigantea* foraged in the wild or cultivated in Yunnan showed low radioactivity of the radiocaesium isotopes—^134^Cs was undetected and ^137^Cs activity concentrations were in the range from <7.0 to 6.8 ± 1.3 Bq kg^−1^ db in the caps and from <4.8 to 7.9 ± 1.7 Bq kg^−1^ db in the stipes (Falandysz et al. 2015b). In a recent paper available in Chinese, data on the activity concentrations of ^137^Cs and ^40^K in some fungi collected from the Mangshi area in southwestern region of Yunnan in 2012–2013 were reported. They, like species in our study, showed low activity concentrations of ^137^Cs. In details, they contained ^137^Cs (adapted data) at 3.8 ± 0.4 Bq kg^−1^ db in *B. aereus*, from 0.73 ± 0.22 to 1.9 ± 0.3 Bq kg^−1^ db in *B. brunneissimus*, from 0.63 ± 0.20 to 6.5 ± 0.6 Bq kg^−1^ db in *B. edulis* and at 1.8 ± 0.3 Bq kg^−1^ db in *B. obscureumbrinus* (Tuo et al. 2014).

### ^40^K activity concentrations in *Boletus* species

Potassium (K) is an important macronutrient for fungi and is the most abundant metallic element in the fruiting bodies. Natural K (^39,40,41^K) contains the long-lived radioactive (^40^K) at 0.012%. The median value of ^40^K activity concentration in the caps for a whole collection of *Boletus* spp. (83 composite samples with data) was at 1300 Bq kg^−1^ db, and the arithmetic mean and standard error were at 1300 ± 200 Bq kg^−1^ db and range was 420–1900 Bq kg^−1^ db. The stipes were poorer in ^40^K than the caps, the median value for a whole collection (81 composite samples with data) was 960 Bq kg^−1^ db, and the arithmetic mean and standard error were at 1000 ± 260 Bq kg^−1^ db and range was 240–1800 Bq kg^−1^ db. Consequently, the median value of the index Q_C/S_ for ^40^K in fungi of the genus *Boletus* in this study (75 composite samples with data) was at 1.2 (the arithmetic mean and standard error values were 1.3 ± 0.3 and range 0.44–4.2).

In our studies of fungi from China, the nuclide ^40^K was at low activity concentrations in sclerotia of *W. extensa* (range between <50 and <83 Bq kg^−1^ db), while in *M. gigantea*, the caps range from 820 ± 150 to 3300 ± 260 Bq kg^−1^ db and the stipes from 770 ± 74 to 1200 ± 100 Bq kg^−1^ db (Falandysz et al. 2015b; Wang et al. 2015). ^40^K in *B. aereus*, *B. brunneissimus, B. edulis*, and *B. obscureumbrinus* from the Mangshi region in Yunnan in the study by Tuo et al. (2014) was in range 610 ± 4s1–1100 ± 71 Bq kg^−1^ db, in which values agree with data from present study (Table 1).

### Probable intake and effective radiation doses from ^137^Cs and ^40^K

To evaluate a possible risk arising from the radioactivity for the inhabitants of the Yunnan region eating mushrooms, the annual effective dose from internal exposure to ^137^Cs and ^40^K from fungal meals has been assessed (Table 2). Blanching (boiling for a short time) or frying of mushrooms can decrease content of ^137^Cs (especially blanching) in a cooked product (Skibniewska and Smoczyński 1999; Steinhauser and Steinhauser 2016). When traditionally cooking mushrooms using a wok pan, the juice generated is not discarded. Hence, we assumed that no leaching of ^137^Cs and ^40^K out of a mushroom meal take place when traditionally cooking the *Boletus* fungi using a wok pan in Yunnan.

**Table 2:** Estimated annual effective radiation dose (μSv) for the internal exposure of ^137^Cs and ^40^K in mushrooms of the genus *Boletus* species from the Yunnan and Sichuan Provinces, China

Mushroom and location and year of collection	^137^Cs (μSv/annum)		^40^K (μSv/annum)	
	Fruiting bodies		Fruiting bodies	
	Caps	Stipes	Caps	Stipes
*Boletus aereus* Fr. ex Bull				
Yongren, Chuxiong; 2012	<0.0062	<0.0051	0.74 ± 0.08	0.51 ± 0.06
Dongshan, Wenshan; 2012	0.029 ± 0.005	0.014 ± 0.003	0.99 ± 0.19	0.54 ± 0.09
*Boletus auripes* Peck				
Yimen, Yuxi; 2011	0.010 ± 0.003	0.0075 ± 0.0022	0.68 ± 0.09	0.38 ± 0.07
Wuding, Chuxiong; 2011	0.014 ± 0.003	0.011 ± 0.002	0.87 ± 0.11	0.52 ± 0.10
*Boletus bicolor* Peck				
Jiangchuan, Yuxi; 2012	0.011 ± 0.002	0.011 ± 0.002	0.68 ± 0.07	0.74 ± 0.07
Dayingjie, Yuxi; 2013	0.0068 ± 0.0022	0.0064 ± 0.0014	0.39 ± 0.09	0.29 ± 0.06
Dayingjie, Yuxi; 2014	0.027 ± 0.004	0.013 ± 0.0026	0.81 ± 0.12	0.87 ± 0.16
*Boletus brunneissimus* Chiu				
Anning, Kunming; 2012	0.017 ± 0.003	0.0070 ± 0.0018	0.74 ± 0.09	0.62 ± 0.06
*Boletus calopus* Fr				
Malong, Qujing; 2013	0.0088 ± 0.0014	0.0049 ± 0.0012	0.62 ± 0.05	0.48 ± 0.47
*Boletus edulis* Bull: Fr				
Jinning, Kunming; 2011	0.0062 ± 0.0044	0.012 ± 0.002	0.68 ± 0.09	0.56 ± 0.08
Anning, Kunming; 2012	0.018 ± 0.003	0.014 ± 0.003	WD	WD
Shilin, Kunming; 2012	0.017 ± 0.001	0.012 ± 0.002	0.87 ± 0.07	0.62 ± 0.06
Yimen, Yuxi; 2011	0.013 ± 0.001	0.0065 ± 0.0013	0.46 ± 0.05	0.22 ± 0.04
Yimen, Yuxi; 2012	0.0070 ± 0.0016	0.0072 ± 0.0016	0.50 ± 0.05	0.31 ± 0.04
Yimen, Yuxi; 2012	0.011 ± 0.002	0.0060 ± 0.0014	0.68 ± 0.06	0.74 ± 0.04
Nanhua, Chuxiong; 2011	0.012 ± 0.003	0.0092 ± 0.0027	0.93 ± 0.11	0.52 ± 0.09
Nanhua, Chuxiong; 2011	0.0061 ± 0.0018	0.014 ± 0.003	0.74 ± 0.07	0.68 ± 0.07
Nanhua, Chuxiong; 2013	0.0047 ± 0.0130	<0.0031	WD	WD
Midu, Dali; 2012	0.013 ± 0.003	0.017 ± 0.003	0.74 ± 0.07	0.53 ± 0.06
Heqing, Dali; 2012	0.0061 ± 0.0018	<0.0043	0.81 ± 0.07	0.53 ± 0.06
Longyang region, Baoshan; 2012	0.0098 ± 0.0018	0.0052 ± 0.0012	WD	WD
Dongshan, Wenshan; 2012	<0.0059	<0.0042	0.74 ± 0.07	0.58 ± 0.05
*Boletus reticuloceps*				
Weixi, Diqing; 2012	0.014 ± 0.003	0.011 ± 0.002	0.87 ± 0.07	0.49 ± 0.05
*Boletus erythropus* Fr.				
Malong, Qujing; 2013	0.011 ± 0.002	0.0081 ± 0.0020	0.62 ± 0.06	0.53 ± 0.09
*Boletus ferrugineus* Schaeff				
Pudacuo, Diqing; 2012	0.018 ± 0.004	WD	0.60 ± 0.07	WD
Lanping, Nujiang; 2012	0.020 ± 0.001	0.026 ± 0.001	0.26 ± 0.03	0.32 ± 0.04
Wuding, Chuxiong; 2011	<0.0048	<0.0049	0.74 ± 0.07	0.62 ± 0.08
Kunming city; 2011	<0.0043	<0.0042	0.68 ± 0.06	0.58 ± 0.06
Shilin, Kunming; 2012	0.0073 ± 0.0018	0.0051 ± 0.0017	0.45 ± 0.04	0.43 ± 0.04
Yimen, Yuxi; 2012	0.011 ± 0.003	0.016 ± 0.003	0.55 ± 0.08	0.44 ± 0.07
*Boletus fulvus* Peck				
Midu, Dali; 2012	0.012 ± 0.002	0.0068 ± 0.0018	0.62 ± 0.07	0.50 ± 0.06
*Boletus griseus* Frost.				
Kunming city; 2011	0.0095 ± 0.0021	0.021 ± 0.003	0.99 ± 0.07	0.93 ± 0.09
Anning, Kunming; 2012	0.020 ± 0.003	<0.0062	0.81 ± 0.09	0.87 ± 0.08
Shilin, Kunming; 2012	0.0072 ± 0.0020	<0.0046	1.1 ± 0.1	0.87 ± 0.06
Longyang region, Baoshan; 2012	0.011 ± 0.002	0.011 ± 0.002	1.1 ± 0.1	1.1 ± 0.1
Jiangchuan, Yuxi; 2012	0.0088 ± 0.0020	<0.0064	0.93 ± 0.07	0.81 ± 0.07
Midu, Dali; 2012	0.012 ± 0.002	<0.0065	0.93 ± 0.08	0.93 ± 0.08
Malong, Qujing; 2012	<0.0062	0.017 ± 0.003	0.87 ± 0.08	0.81 ± 0.09
Lufeng, Chuxiong; 2013	0.0075 ± 0.0014	0.0052 ± 0.0013	0.93 ± 0.05	0.74 ± 0.05
*Boletus impolitus* Fr.				
Kunming city; 2011	0.010 ± 0.002	0.017 ± 0.003	0.87 ± 0.07	0.55 ± 0.07
Yimen, Yuxi; 2012	0.014 ± 0.003	0.020 ± 0.003	0.68 ± 0.07	0.58 ± 0.08
*Boletus luridus* Schaoff. Fr.				
Yuanmou, Chuxiong; 2012	0.013 ± 0.002	0.014 ± 0.003	0.93 ± 0.09	0.53 ± 0.08
Midu, Dali; 2012	0.0096 ± 0.0022	0.012 ± 0.002	0.93 ± 0.07	0.81 ± 0.07
Shilin, Kunming; 2012	0.047 ± 0.003	0.013 ± 0.001	0.62 ± 0.07	0.15 ± 0.05
*Boletus magnificus* Chiu.				
Jiangchuan, Yuxi; 2012	0.0051 ± 0.0016	<0.0052	0.68 ± 0.07	0.56 ± 0.06
Yimen, Yuxi; 2012	0.0079 ± 0.0021	0.0077 ± 0.0016	0.87 ± 0.07	0.46 ± 0.05
Yimen, Yuxi; 2012	<0.0068	0.013 ± 0.002	0.93 ± 0.09	0.60 ± 0.11
Midu, Dali; 2012	0.0074 ± 0.0017	WD	0.51 ± 0.06	WD
Dayingjie, Yuxi; 2014	0.011 ± 0.002	0.011 ± 0.002	0.93 ± 0.07	0.68 ± 0.07
*Boletus obscureumbrinus* Hongo				
Simao region, Puer; 2013	0.014 ± 0.003	<0.0061	0.99 ± 0.08	0.93 ± 0.07
Simao region, Puer; 2013	0.020 ± 0.003	0.0098 ± 0.0021	0.87 ± 0.09	0.87 ± 0.07
*Boletus pallidus* Frost.				
Anning, Kunming; 2012	0.013 ± 0.002	<0.0065	1.1 ± 0.1	0.62 ± 0.08
*Boletus purpureus* Fr.				
Yimen, Yuxi; 2012	0.0088 ± 0.0021	0.0074 ± 0.0022	WD	WD
Yimen, Yuxi; 2012	0.012 ± 0.002	0.011 ± 0.003	1.2 ± 0.1	1.1 ± 0.1
Yunlong, Dali; 2012	0.016 ± 0.003	0.0079 ± 0.0025	0.81 ± 0.11	0.54 ± 0.09
Lanping, Nujiang; 2012	0.0081 ± 0.0022	0.013 ± 0.002	0.74 ± 0.07	0.52 ± 0.07
*Boletus reticuloceps* Zang et al.				
Pudacuo, Diqing; 2012	0.017 ± 0.001	0.013 ± 0.002	0.74 ± 0.05	0.43 ± 0.06
*Boletus sinicus* W.F. Chiu				
Malong, Qujing; 2013	0.0082 ± 0.0020	0.0086 ± 0.0018	0.93 ± 0.07	0.74 ± 0.06
Jiulongchi, Yuxi; 2013	0.0094 ± 0.0021	0.0042 ± 0.0012	0.87 ± 0.06	0.68 ± 0.04
*Boletus speciosus* Forst.				
Anning, Kunming; 2012	<0.0072	0.010 ± 0.002	0.62 ± 0.07	0.45 ± 0.05
Anning, Kunming; 2012	0.027 ± 0.004	0.013 ± 0.002	0.93 ± 0.11	0.62 ± 0.07
Yimen, Yuxi; 2012	0.0088 ± 0.0020	0.012 ± 0.002	0.68 ± 0.07	0.45 ± 0.07
Longyang region, Baoshan; 2012	0.018 ± 0.004	0.012 ± 0.002	0.87 ± 0.11	0.52 ± 0.07
Yuanmou, Chuxiong; 2012	0.0079 ± 0.0017	0.0061 ± 0.0014	0.68 ± 0.05	0.43 ± 0.04
Huize, Qujing; 2013	0.0086 ± 0.0020	<0.0034	0.62 ± 0.06	0.47 ± 0.04
*Boletus tomentipes* Earle				
Eshan, Yuxi; 2011	<0.0057	0.012 ± 0.002	0.99 ± 0.07	0.87 ± 0.06
Eshan, Yuxi; 2011	0.011 ± 0.002	0.0055 ± 0.0017	0.74 ± 0.06	0.74 ± 0.06
Eshan, Yuxi; 2011	0.0090 ± 0.0016	0.0059 ± 0.0016	0.81 ± 0.07	0.74 ± 0.05
Yimen, Yuxi; 2011	0.012 ± 0.002	0.013 ± 0.002	0.99 ± 0.07	0.81 ± 0.06
Yimen, Yuxi; 2011	0.0075 ± 0.0018	0.013 ± 0.002	0.74 ± 0.06	0.81 ± 0.07
Simao region, Puer; 2011	0.018 ± 0.003	0.014 ± 0.003	1.2 ± 0.1	0.87 ± 0.07
Nanhua, Chuxiong; 2011	0.0092 ± 0.0018	0.013 ± 0.002	0.74 ± 0.07	0.81 ± 0.07
Gejiu, Honghe; 2012	0.018 ± 0.003	0.0070 ± 0.0016	0.93 ± 0.07	0.81 ± 0.05
Shangri-la, Diqing; 2011	0.0081 ± 0.0017	0.0086 ± 0.0020	0.87 ± 0.05	0.81 ± 0.06
Shiping, Honghe; 2012	0.0091 ± 0.0021	0.0043 ± 0.0010	0.81 ± 0.06	0.58 ± 0.04
Dechang, Sichuan; 2012	0.0062 ± 0.0021	0.011 ± 0.002	1.1 ± 0.1	1.1 ± 0.1
Panzhihua, Sichuan; 2012	0.0069 ± 0.0017	0.012 ± 0.002	0.93 ± 0.06	0.99 ± 0.06
Heqing, Dali; 2012	0.0068 ± 0.0014	0.0056 ± 0.0014	0.68 ± 0.05	0.62 ± 0.05
*Boletus umbriniporus* Hongo				
Yimen, Yuxi; 2011	0.0064 ± 0.0018	0.013 ± 0.003	0.68 ± 0.07	0.39 ± 0.09
Yimen, Yuxi; 2012	0.018 ± 0.003	0.0094 ± 0.0021	0.93 ± 0.09	0.81 ± 0.09
Huangcaoba, Yuxi; 2011	0.0083 ± 0.0016	<0.0047	0.56 ± 0.05	0.56 ± 0.06
Longyang region, Baoshan; 2012	0.0085 ± 0.0017	0.010 ± 0.002	0.87 ± 0.06	0.60 ± 0.07
Midu, Dali; 2012	0.017 ± 0.001	<0.0051	0.81 ± 0.06	0.68 ± 0.06
Shiping, Honghe; 2012	0.010 ± 0.002	0.011 ± 0.002	0.61 ± 0.06	0.41 ± 0.05
Yuanmou, Chuxiong; 2011	0.012 ± 0.002	0.021 ± 0.003	0.81 ± 0.07	0.74 ± 0.07
Huize, Qujing; 2013	0.011 ± 0.002	0.0094 ± 0.0017	0.74 ± 0.06	0.55 ± 0.05
Nanhua, Chuxiong; 2013	0.011 ± 0.002	0.0068 ± 0.0013	0.74 ± 0.06	0.58 ± 0.05

see Fig. 1; number of individuals

*WD* without data

The annual effective radiation dose figures due to ^137^Cs intake with 1 kg of fresh mushrooms per annum depending on species and location range for ^137^Cs from around <0.0031 to 0.047 ± 0.003 μSv, while those for ^40^K from around 0.22 ± 0.04 to 1.2 ± 0.1 μSv (Table 2). If considering a maximal figure for mushrooms consumption by some locals in Sichuan and Yunnan with high intake (up to around 30 kg fresh biomass per annum), the figures above have to be multiplied by 20–30, i.e., can be for ^137^Cs from around 0.062–0.093 to 0.94–1.4 μSv, while those for ^40^K from around 4.4–6.6 to 24 to 36 μSv. They both figures obtained on the annual effective radiation dose for ^137^Cs and ^40^K contained in mushrooms of the genus *Boletus* in Sichuan and Yunnan, and summed up are very low. Considered a potential, the effective dose from the ^40^K contained in the *Boletus* mushrooms from the Yunnan was an order of magnitude greater than from ^137^Cs.

Summing up, the activity concentrations of ^137^Cs in mushrooms of the genus *Boletus* foraged in 2011–2014 in the Sichuan and Yunnan Provinces of China are very low and mushroom meals contribute there at very low rate to the annual effective radiation dose for individuals. The activity concentrations of ^137^Cs in mushrooms of the genus *Boletus* in the Sichuan and Yunnan are around 100-fold below activity concentrations of ^40^K. Considered a potential, the effective dose from the ^137^Cs and ^40^K, separately and combined, contained in the *Boletus* mushrooms from the Yunnan, was very low and it was an order of magnitude greater from the natural nuclide ^40^K than ^137^Cs.
